# Accelerated ^19^F·MRI Detection of Matrix Metalloproteinase-2/-9 through Responsive Deactivation of Paramagnetic Relaxation Enhancement

**DOI:** 10.1155/2019/4826520

**Published:** 2019-02-28

**Authors:** Henryk M. Faas, James L. Krupa, Alexander J. Taylor, Francesco Zamberlan, Christopher J. Philp, Huw E. L. Williams, Simon R. Johnson, Galina E. Pavlovskaya, Neil R. Thomas, Thomas Meersmann

**Affiliations:** ^1^Sir Peter Mansfield Imaging Centre, University of Nottingham, Nottingham NG7 2RD, UK; ^2^Division of Clinical Neuroscience, School of Medicine, University of Nottingham, Nottingham NG7 2UH, UK; ^3^Centre for Biomolecular Sciences, School of Chemistry, University of Nottingham, Nottingham NG7 2RD, UK; ^4^Respiratory Medicine and Biomedical Research Centre, School of Medicine, University of Nottingham, Nottingham NG7 2UH, UK; ^5^Department of Electrical and Electronic Engineering, University of Nottingham, Ningbo 315100, China

## Abstract

Paramagnetic gadolinium ions (Gd^III^), complexed within DOTA-based chelates, have become useful tools to increase the magnetic resonance imaging (MRI) contrast in tissues of interest. Recently, “on/off” probes serving as ^19^F·MRI biosensors for target enzymes have emerged that utilize the increase in transverse (*T*_2_^*∗*^ or *T*_2_) relaxation times upon cleavage of the paramagnetic Gd^III^ centre. Molecular ^19^F·MRI has the advantage of high specificity due to the lack of background signal but suffers from low signal intensity that leads to low spatial resolution and long recording times. In this work, an “on/off” probe concept is introduced that utilizes responsive deactivation of paramagnetic relaxation enhancement (PRE) to generate ^19^F longitudinal (*T*_1_) relaxation contrast for accelerated molecular MRI. The probe concept is applied to matrix metalloproteinases (MMPs), a class of enzymes linked with many inflammatory diseases and cancer that modify bioactive extracellular substrates. The presence of these biomarkers in extracellular space makes MMPs an accessible target for responsive PRE deactivation probes. Responsive PRE deactivation in a ^19^F biosensor probe, selective for MMP-2 and MMP-9, is shown to enable molecular MRI contrast at significantly reduced experimental times compared to previous methods. PRE deactivation was caused by MMP through cleavage of a protease substrate that served as a linker between the fluorine-containing moiety and a paramagnetic Gd^III^-bound DOTA complex. Ultrashort echo time (UTE) MRI and, alternatively, short echo times in standard gradient echo (GE) MRI were employed to cope with the fast ^19^F transverse relaxation of the PRE active probe in its “on-state.” Upon responsive PRE deactivation, the ^19^F·MRI signal from the “off-state” probe diminished, thereby indicating the presence of the target enzyme through the associated negative MRI contrast. Null point ^1^H·MRI, obtainable within a short time course, was employed to identify false-positive ^19^F·MRI responses caused by dilution of the contrast agent.

## 1. Introduction

The measurement of enzyme activity *in vivo* is a major challenge for the development of enzyme-specific chemical probes and will facilitate a deeper understanding of the role of enzymes in biological processes and facilitate drug discovery, synthetic biology, and metabolic engineering research [1]. Matrix metalloproteinases (MMPs), a subclass of proteases, are zinc(II)-dependent enzymes that typically possess two Zn^II^ ions, one for structural purposes and the other critical for catalysis. In humans, there are 24 different genes which code for 23 different MMPs, with the first and last gene coding for the same MMP [2]. Expressed in a latent form prior to activation extracellularly, MMPs are an attractive drug target, due to their selective activation location outside the cell. MMPs remodel the extracellular matrix (ECM) and also play key roles in a range of physiological processes, including wound healing [3], organogenesis, and modulation of inflammatory processes. MMPs are also involved in pathological processes such as chronic obstructive pulmonary disease (COPD) [4] and can be used as biomarkers for determining stages of cancer, where the levels of MMPs are correlated to metastatic potential [5].

Chemical probes to detect MMPs have been developed in the field of fluorescent microscopy. Förster resonance energy transfer- (FRET-) based MMP probes have been available since the early 1990s [6], but *in vivo* imaging applications are hampered by the limited tissue penetration of the short wavelength light, inherently used for excitation and emission in fluorescent techniques. As a result, *ex vivo* studies have been performed, for example, using probes to measure serum MMP levels [7]. An alternative and noninvasive imaging modality is magnetic resonance imaging (MRI). Chemical probes or responsive contrast agents have been previously developed for use in ^1^H·MRI, providing information on a range of catalytic [8] and noncatalytic processes such as monitoring pH [9], redox dysregulation [10], and levels of metal ions such as zinc [11], calcium [12], and copper [13]. However, to date, there have only been a small number of examples of MMP MRI probes [14]. Notable examples include iron oxide nanoparticles which have been used to show changes in *T*_2_ relaxation times upon conjugation with MMPs [15] and contrast agents which use the concept of a solubility switch in which the *T*_1_ value is altered due to the precipitation resulting from MMP ligand cleavage [16].

A key hurdle in the successful application of ^1^H·MMP probes is the confounding background signal from endogenous water in the body, which makes contrast determination difficult. To overcome this problem, efforts have focussed on ^19^F, for which there is little detectable ^19^F background signal in the body (except for bones, teeth and calcified tissue), leading to excellent specificity [17]. This is an important requirement for a molecular probe and a key advantage over ^1^H·MRI [18]. An exciting approach to harness the effect of paramagnetic Gd^III^ upon ^19^F relaxation was proposed by Mizukami et al. to study the enzyme activity using a peptide of four amino acid residues that acts as a substrate for caspase-3. A trifluoro aryl ether at the carboxy terminus of the caspase-3 peptide substrate was linked with a Gd^III^-DOTA chelate at its amine terminus [19]. In the initial uncleaved form, the fluorine signal is weak due to severe line broadening by fast transverse relaxation (i.e., short *T*_2_ times). However, in the presence of the specific enzyme, the linker was cleaved and the distance between the Gd^III^ DOTA and fluorine increased, causing a reduction of the paramagnetic influence on fluorine. The decreased transverse relaxation led to an increase of the ^19^F signal due to line narrowing. The line-narrowing response of the probe molecule—or biosensor molecule—produces detectable MRI signal in regions where the biosensor is co-located with the enzyme target. Using and validating this concept, further ^19^F biosensor molecules were designed to probe for *β*-galactosidase and *β*-lactamase activities [20–22]. Previous developments also include a dual fluorescence ^19^F probe [23] and a dual ^1^H-^19^F·MRI probe which in its precleaved form contained a labile carbamate with a trifluoromethyl group in close proximity to a chelated Gd^III^ ion [24]. The ^1^H-^19^F probe was then activated by *β*-galactosidase, which cleaved the carbamate resulting in the release of the ^19^F source, in turn increasing the ^19^F·MRI signal intensity. For MMP-2 detection, equivalent to the present work, an “off/on” probe with nine equivalent ^19^F atoms as signal source has been reported by Yue et al., which utilizes this effect of cleavage to alter the ^19^F transverse relaxation properties [25, 26].

Although significant progress has been made with nonspecific ^19^F·MRI tracers for cell labelling, including clinical applications [27, 28], the use of specifically targeted ^19^F·MRI probes for molecular imaging, in particular for *in vivo* studies, is frustrated by inadequate ^19^F·MRI signal intensity [29]. The MMP biosensor concept described above utilizes reduced transverse (*T*_2_ or *T*_2_^*∗*^) relaxation; however, the longitudinal relaxation is also reduced, and long *T*_1_ times diminish the amount of signal averaging that is feasible within the time span of typical *in vivo* MRI experiments.

In this work, we have explored a modified MMP ^19^F biosensor detection protocol where the observation concept is turned around by detecting the intact biosensor instead of the cleaved molecule. The short ^19^F·*T*_1_ relaxation time of the intact biosensor molecule is utilized for paramagnetic relaxation enhancement (PRE) [30–32] to improve the ^19^F·MRI signal to noise ratio (S/N) through rapid signal averaging. MRI protocols are adjusted to handle the fast transverse relaxation of PRE-activated biosensors. Responsive cleavage of the biosensor by the target protein causes PRE to be “switched off” and the MRI signal to disappear. The resulting negative MRI contrast is demonstrated to serve as an indicator for the presence of MMP biomarker molecules.

## 2. Materials and Methods

### 2.1. Synthesis of the Biosensor

**Figure 1:**
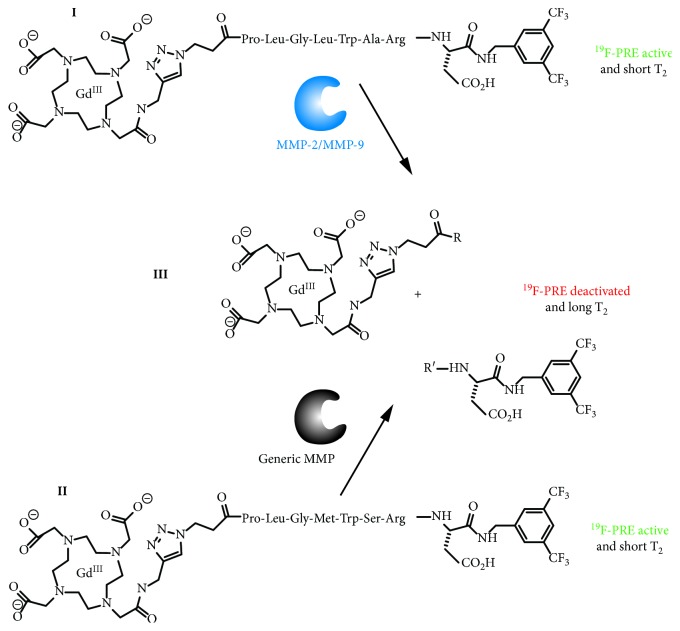
Sketch of MMP responsive line narrowing and PRE deactivation. (**I**) Gd^III^ PRE-activated ^19^F probe for sequence-specific MMP-9/12, (**II**) Gd^III^ PRE-activated ^19^F broad-range probe. Before cleavage, the Gd^III^ is in close proximity to the ^19^F causing short *T*_2_ times that can be observed as ^19^F·NMR line broadening. However, a strong paramagnetic relaxation enhancement (PRE) effect is also present that enables rapid signal averaging due to short *T*_1_ times. After cleavage (**III**), the distance *r* between the Gd^III^ and the ^19^F moieties substantially increases, leading to line narrowing but also to the deactivation of ^19^F·PRE due to the 1/*r* ^6^ dependence of paramagnetic relaxation.

To advance the concept of responsive PRE deactivation, two different “on/off” ^19^F·MRI probes sensitive to MMP cleavage have been synthesized using a short customisable synthetic route. The probes can be split into three segments: (1) the fluorine-containing moiety, which delivers the signal; (2) the paramagnetic moiety for PRE, which consists of a Gd^III^-bound DOTA-complex; (3) the protease substrate which links together the other two constituents and can be tailored to serve as substrate to specific target for MMPs (see Figure 1 and also Supporting Materials S1 for selected sequences). The main focus of the current work is on the concept of molecular contrast through responsive PRE deactivation using molecular probes that are mechanistically similar to the MMP-2 selective probe previously utilized by Yue et al. for *T*_2_^*∗*^ contrast [25]. Some changes in the probe structure and its synthesis have been made compared to [25]. In the previous work, a PEG linker was incorporated for solubility of the fluorine-containing moiety and was attached to the MMP substrate peptide via Michael addition of maleimide to the thiol of the cysteine-containing substrate peptide. In the current work, this type of linker, known to be labile due to thiol exchange *in vivo* [33], was avoided, and the fluorine moiety was directly incorporated as amino acid side chain in the peptide sequence; furthermore, Gd^III^-bound DOTA-complex was linked to the peptide sequence using a stable 1,4-triazole formed using “click” chemistry. The molecule in this work is soluble in 9 : 1 (v/v) water/acetonitrile up to at least 1.2 mM concentration without a PEG linker, and the solvent was acceptable for the proof of concept work and tolerated by the enzyme. Furthermore, Yue et al. used peptide-coupling chemistry to attach an octadentate Gd^III^-DOTA chelator as the final unit to their peptide. This synthesis required 30 synthetic steps, two of which required purification by high-performance liquid chromatography (HPLC). The probes in the current work required 27 synthetic steps, with purification via HPLC reserved for only the final step adding some efficiency. The synthesis is described in detail in Supplementary Materials S1–S3.

### 2.2. MMP Preparation

Recombinant human carrier-free MMP-1, MMP-2, MMP-9, and MMP-12 (R&D Systems, Minneapolis, Minnesota) were diluted upon receipt to 75 *µ*g/ml in 50 mM Tris, 10 mM·CaCl_2_, 150 mM·NaCl, 0.05% (w/v) Brij 35, and pH 7.5. MMPs were activated by incubation with 4-aminophenylmercuric acetate (APMA) at 100 mM in dimethyl sulfoxide (DMSO) (Sigma Aldrich, UK) was added to a final concentration of 1 mM. Activation incubation times at 310 K were 1 hour for MMP1-1 and MMP-2 and 24 hours for MMP-9 and MMP-12. Following incubation, enzymes were aliquoted and frozen at 193 K until required. A 5 *μ*l aliquot was thawed and added to 600 *µ*l of the biosensor solution, immediately prior to the NMR experiments.

### 2.3. Enzyme Activity Time Course Observed through ^19^F·NMR Line Shape at 14.1 Tesla

**Figure 2:**
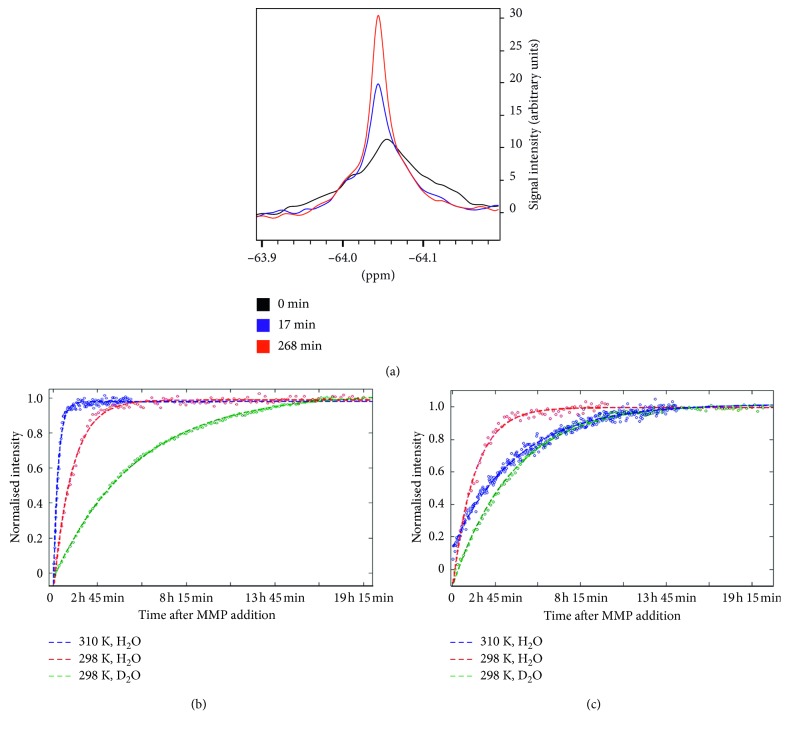
^19^F·NMR spectroscopy of MMP-mediated biosensor cleavage at 14.1 Tesla. (a) ^19^F·NMR lineshape of 0.1 mM-specific MMP-2/-9 probe as a function of time at 298 K. After MMP-9 was added at *t* = 0, the NMR signal of the probe showed an increase in the amplitude and sharpening of the peak. (b) Time course of NMR signal intensity (amplitude) of the specific MMP-2/-9 probe (0.1 mM) as a function of time after MMP incubation under different conditions: at 298 K in pure D_2_O, at 298 K in v/v 9 : 1 H_2_O/D_2_O, and at 310 K in v/v 9 : 1 H_2_O/D_2_O. The fastest reaction rate was observed at physiological temperatures (310 K). (c) Time course of NMR signal intensity (amplitude) of the broad-range probe with MMP-9 added at *t* = 0. For the broad-range probe, the reaction rate was fastest at room temperature (298 K), in contrast to the optimal conditions for the specific MMP-2/-9 probe.

^19^F·NMR/MRI·MMP probe (0.1 mM in 9 : 1 (v/v) H_2_O/D_2_O) was incubated with MMP-9 (5 *µ*L, at 10 *µ*g/mL, final concentration 83 ng/mL) in a total volume 600 *µ*L adjusted to pH 7.5. The time point *t* = 0 for each time course was right after MMP addition; subsequently, the sample was reintroduced into the NMR magnet and kept at the temperature specified. The biosensor molecule (Figure 1) results to a single peak at –64 ppm (relative to CFCl_3_ at 0 ppm). The ^19^F·NMR lineshape measurements (Figure 2) were obtained in a 5 mm sample diameter high-resolution probe head with ^19^F inner coil for acquisition (^1^H outer coil for decoupling—not used) tuned to the ^19^F frequency of 564.6 MHz using a Bruker Avance III 600 MHz (14.1 Tesla) spectrometer. The fluorine linewidth (FWHM) was used as a measure of the transverse relaxation, *T*_2_^*∗*^ [34]. Measurements were taken at a temperature of either 298 K or 310 K as indicated in the figure and main text. The time dependence of the signal intensity was analyzed using Lambert functions for a Michaelis-Menten fit to obtain the enzyme efficiency parameter listed in Table 1.

### 2.4. ^19^F·NMR *T*_1_ Relaxation Measurements at 14.1 Tesla

Longitudinal relaxation rates (*T*_1_) for the fluorinated molecular probes were determined in an inversion recovery experiment at 298 K with 14 to 28 inversion recovery times and a repetition time (TR) of up to 8 seconds to insure full magnetization recovery (Table 2). NMR spectrometer and probe head were identical to those described above the linewidth measurements.

### 2.5. ^19^F·NMR *T*_1_ and *T*_2_ Relaxation Measurements at 9.4 Tesla

**Figure 3:**
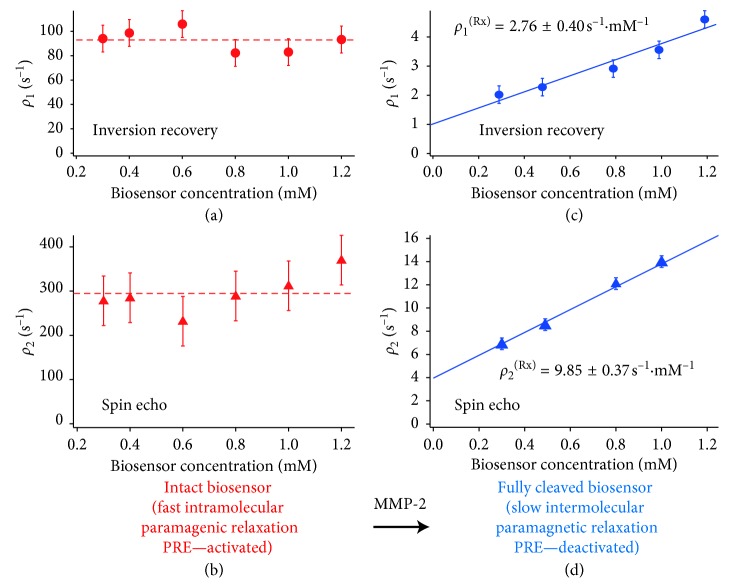
Dependence of ^19^F relaxation rates *ρ*_1_ = *T*_1_^−1^ and *ρ*_2_ = *T*_2_^−1^ on the biosensor concentration [Rx]. Measurements taken at 9.4 Tesla and 293 K using the MMP-2/-9 specific biosensor diluted in 9 : 1 v/v H_2_O/acetonitrile mixture. (a) ^19^F relaxivity *ρ*_1_/[Rx] measurement of the intact biosensor revealed no detectable concentration dependence of ^19^F longitudinal relaxation with an approximate average value of *ρ*_1_ = 93 s^−1^·ms (red dashed line). (b) The transverse relaxation rate of the intact biosensor also exhibited little dependence on [Rx] with an average of *ρ*_2_ = 298 s^−1^·ms (red dashed line). (c, d) After MMP-2 responsive biosensor cleavage, a strongly reduced and concentration [Rx]-dependent longitudinal and transverse relaxation rates (*ρ*_1_ and *ρ*_2_, (c) and (d), respectively) was observed. The solid blue lines in (c) and (d) show data fitting for relaxivity *ρ*/[Rx] defined by a concentration [Rx]-dependent part *ρ* ^[Rx]^ and a concentration independent offset *ρ* ^0^ (Table 3 for determined values).

All bulk relaxation measurements (i.e., without spatial resolution) at 9.4 Tesla were obtained for samples contained within a single 5 mm NMR sample tube located in the centre (i.e., axial location) of a 30 mm (sample diameter) ^19^F microimaging probe head (Bruker) tuned to 376.5 MHz using a Bruker Avance III 400 MHz (9.4 Tesla) spectrometer and microimaging system. The temperature for all 9.4 Tesla measurements was 293 K. The *T*_1_ and *T*_2_ relaxation times of intact (PRE activated) ^19^F biosensor dissolved at concentrations ranging from 0.3–1.2 mM in 9 : 1 (v/v) water/acetonitrile (ACN) were obtained using standard inversion recovery and spin echo experiments, respectively. A 82 *μs* rectangular 90° pulse was used for excitation, and 164 *μs* rectangular 180° pulses was used for inversion and refocusing. The integrated signal intensity and signal heights were obtained from 12–14 inversion recovery and spin echo measurements with a signal averaging of 1024 scans. Likewise, inversion recovery and spin echo measurements were also performed with the cleaved biosensor after completed reaction with the MMP. Recovery times of at least 5 *T*_1_ times were used for all measurements (up to 2.5 s) at the range of recovery and spin echo times that allowed for at least 90 % recovery or 90% decay, respectively. The resulting curves were analysed using Igor Pro 7, and the average from signal intensity curves and integrated intensities are reported in Figure 3.

### 2.6. ^19^F·MRI Protocols at 9.4 Tesla to Monitor MMP Activity

**Figure 4:**
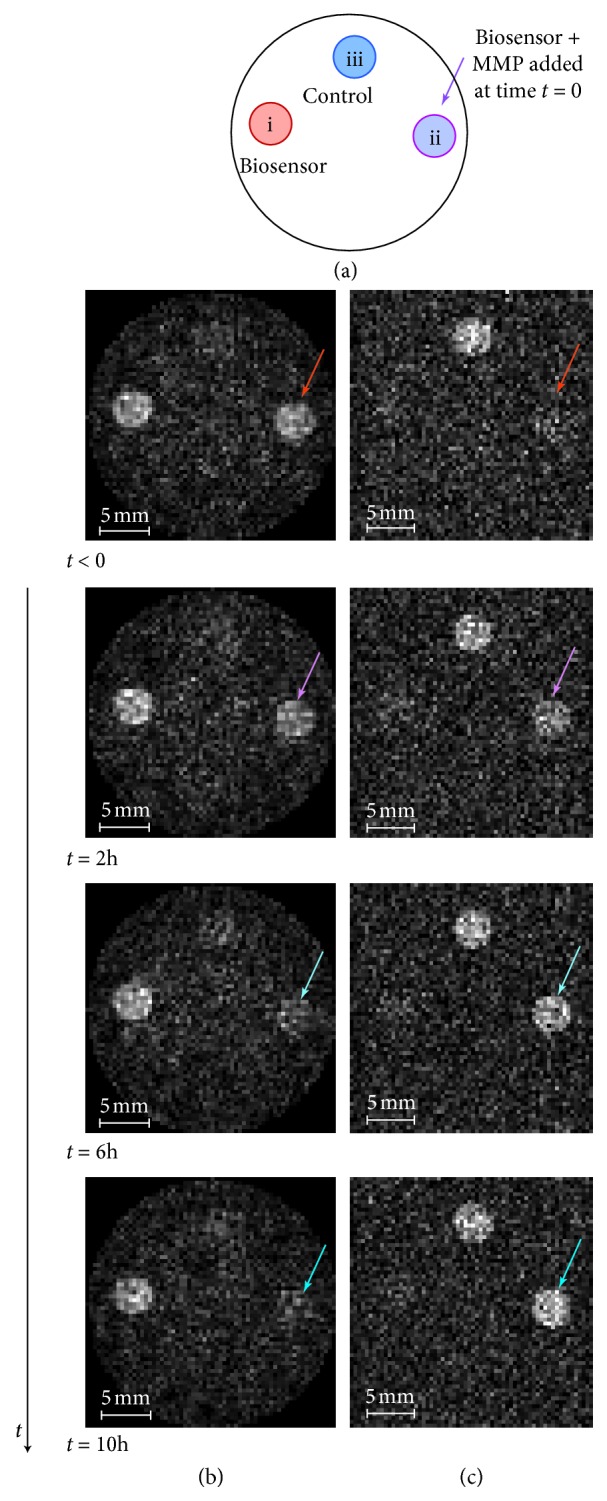
Transverse ^19^F·MRI at 9.4 Tesla and 293 K of three separate 5 mm NMR tubes (located within a 30 mm microimaging probe head), each containing 600 *μ*L of 1.2 mM solution of the respective ^19^F samples in 9 : 1 v/v H_2_O/acetonitrile solution, were used to demonstrate the effect of negative *T*_1_ contrast for molecular imaging. (a) Sketch of the sample (transverse view): (i) 1.2 mM biosensor without MMP. (ii) 1.2 mM biosensor with MMP-2 added at *t* = 0 s. (iii) ^19^F control containing 1.2 mM of tetrafluoroacetate (TFA) and 1.2 mM of Gd-DOTA. Samples were kept at 293 K throughout the reaction. (b) *T*_1_ weighted ^19^F·UTE·MRI at various times with MMP-2 added to sample (ii) at *t* = 0 demonstrating negative contrast with progressing time. (c) *T*_2_^19^F weighted spin echo (SE) MRI demonstrating the effect of positive contrast. Recording time *t* (rounded to the nearest full hour) indicates the beginning of the UTE MRI acquisition (6 min recording time per image) that is followed after completion by the SE MRI (75 min recording per image).

**Figure 5:**
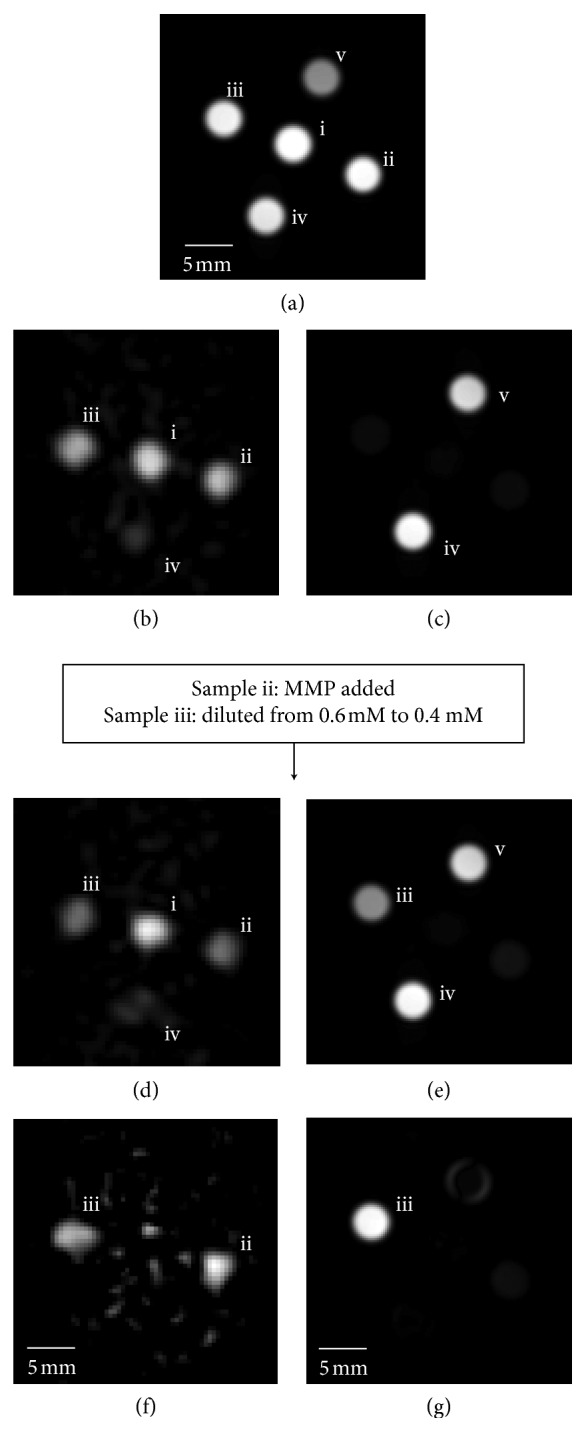
*T*_1_ weighted ^19^F and ^1^H·MRI of samples containing PRE active and MMP-2 cleaved (PRE deactivated) ^19^F biosensors at 9.4 Tesla (transverse view). Samples in five separate NMR tubes located in a 30 mm microimaging probe head at 293 K (i) 0.6 mM biosensor (no MMP-2 added), (ii) 0.6 mM biosensor with MMP-2 added after acquisition of images (b) and (c), (iii) 0.6 mM biosensor, diluted to 0.4 mM concentration after images (b) and (c) acquisition, (iv) 0.4 mM cleaved biosensor (cleaved throughout all images), and (v) PBS solution without any Gd(III) or ^19^F present. (a) ^1^H gradient echo (GE) MRI (of H_2_O) showing all samples. (b) *T*_1_ weighted ^19^F·GE·MRI showing three samples with 0.6 mM PRE active (not cleaved) biosensor probe. (c) ^1^H null point MRI (of H_2_O) with inversion recovery time (TI) set to 220 ms leading to vanishing signal in the samples with PRE active biosensor. (d) *T*_1_ weighted ^19^F·GE·MRI after MMP-2 catalysed cleavage of biosensor in sample (ii) and dilution of sample (iii) to 0.4 mM. (e) ^1^H null point MRI (of H_2_O) with TI = 220 ms revealing the concentration change of sample (iii) (false positive). (f) Difference of *T*_1_ weighted ^19^F·GE·MRI (d subtracted from b). (g) Absolute value of the difference of ^1^H·IR·MRI (e subtracted from c).

All MRI were recorded using a Bruker Avance III 400 MHz (9.4 Tesla) microimaging system with a ^19^F frequency of 376.4 MHz at 293 K. The sample for MRI were contained within three or five (see Figures 4 and 5, respectively) 5mm NMR sample tubes located in a custom-made 30 mm sample holder placed into a 30 mm ^19^F Bruker microimaging probe head tuned to 376.5 MHz. The samples in each tube are described in the figure captions.

2D transverse ultrashort echo time (UTE) ^19^F·MR images (30 mm slice thickness) of a sample containing three 5 mm NMR tubes (Figure 4) were obtained from 202 projections and used a polar undersampling factor of 1. Images were reconstructed to a 64 × 64 data point resolution. The field of view was FOV = 25 mm × 25 mm. A 300 *μ*s Gaussian 90° excitation pulse was used, TE was 191 *μ*s, repetition time was 5 ms, and 360 averages were taken that lead to a total scanning time of 6 min.

2D transverse spin echo ^19^F·MRI (30 mm slice thickness) of sample containing three 5 mm NMR tubes (Figure 4) used a 1 ms Gaussian 90° excitation pulse, 10.29 ms echo time, 200 ms repetition time, and 360 averages. FOV = 30 mm × 30 mm at 32 × 32 point resolution (raw data) recorded at a total acquisition time of 77 min. For processing, a sinebell apodization and zero filling to 64 × 64 data points were applied.

2D transverse gradient echo ^19^F·MRI (50 mm slice thickness) of sample containing five 5 mm NMR tubes (Figure 5) used 873 *μ*s echo time, 20 ms repetition time, and 1536 averages. FOV = 30 mm × 30 mm at 32 × 32 point resolution (raw data) recorded at a total acquisition time of 16 min. For processing, a sinebell apodization and zero filling to 64 × 64 data points were applied.

### 2.7. ^1^H·MRI Protocols at 9.4 Tesla

The 2D gradient echo (GE) ^1^H·MRI (2 mm slice thickness) of sample containing five 5 mm NMR tubes (Figure 5) used a standard GE protocol with 1.4 ms rectangular 90° excitation pulse, 2.5 ms echo time, 2000 ms repetition time, and no signal averaging. FOV = 30 mm × 30 mm at 64 × 64 data point (raw) resolution recorded at a total acquisition time of 2½ min at 293 K. For processing, a sinebell apodization and zero filling to 128 × 128 points were applied.

The 2D null point ^1^H·MRI (2 mm slice thickness) of the sample in Figure 5, containing five 5 mm NMR tubes, used an inversion recovery protocol with 4.84 ms rectangular 180° inversion pulse, 220 ms inversion recovery time, 2.5 ms echo time, 2000 ms repetition time, and no signal averaging. FOV = 30 mm × 30 mm at 64 × 64 data point resolution recorded at a total acquisition time of 2½ min at 293 K. For processing, a sinebell apodization was applied with zero filling to 128 × 128 points.

## 3. Results and Discussion

### 3.1. Following Enzymatic Activity through NMR Linewidth

Intramolecular paramagnetic relaxation, the dominating relaxation mechanism for the ^19^F nuclear spins in the intact biosensor molecule, will be replaced by intermolecular paramagnetic relaxation after biosensor cleavage through MMP (Figure 1). The spatial separation *r* between the ^19^F-containing unit and the paramagnetic Gd DOTA complex will substantially increase by the cleavage, causing a dramatic reduction in the 1/*r* ^6^-dependent paramagnetic relaxation for ^19^F. This means that ^19^F·PRE is effectively “turned off” by the biosensor response to MMP.

The ^19^F·NMR linewidth narrowing, associated with cleavage, as previously utilized by Yue et al. [25, 26], was used in this work for the initial testing of the biosensor and to explore both temperature and also isotope effects on the sensor kinetics. The isotope effect of deuterium on the cleavage reaction is of interest as D_2_O is typically used as a lock solvent in NMR spectroscopy. The MMP-2/-9 selective probe was incubated with APMA- (4-aminophenylmercuric acetate-) activated MMP-9 under three different sets of conditions (i.e., D_2_O at 298 K, H_2_O at 298 K, and H_2_O at 310 K). Before the addition of MMP-9 the ^19^F·NMR peaks observed were broad, with a full width at half maximum (FWHM) of ∼60 Hz (310 K). After addition of MMP-9, a time series of ^19^F·NMR experiments was performed to monitor the progress of the enzymatic reaction. The observed line narrowing with reaction time in Figure 2(a) indicates the progressive cleavage of the biosensor with associated reduction in the transverse paramagnetic relaxation.

The amplitude of the single ^19^F peak at −64 ppm (relative to CFCl_3_ at 0 ppm) was measured and used to plot peak amplitude versus time. Postcleavage, the FWHM was reduced to 19 Hz (310 K), caused by the increase in distance between the Gd^III^ ion and the ^19^F signal source. These experiments were repeated with the broad-range probe under identical conditions, and a FWHM of ∼53 Hz precleavage, and 13 Hz postcleavage (310 K) was observed (not shown).

The time course of the NMR signal under the three different sets of conditions (varying solvent and temperature) is shown for the MMP-2/-9 probe in Figure 2(b) and for the broad-range probe in Figure 2(c). In order to compare the relative conditions and probes, we calculated the specificity constant (enzyme efficiency), *k*_cat_/*K*_M_, for both probes under each set of condition (Table 1). Here, *k*_cat_ is the turnover number (per second) and *K*_M_ the apparent (Michaelis) binding constant. The enzyme efficiency was calculated by mapping the time dependence of amplitude of the signal peak and then applying a Michaelis-Menten based fit using Lambert functions and least squares fitting [35].

At physiological temperatures, the MMP-2/-9 probe was almost completely cleaved within an hour. The enzyme was fivefold more efficient (seen in the change in *k*_cat_/*K*_M_) if the solvent contained 10% v/v D_2_O rather than 100% D_2_O (at 298 K). A similar trend was observed with the broad-range probe, where the enzyme was twice as efficient in 10% v/v D_2_O rather than pure D_2_O. Intriguingly, we observed that, in contrast to the specific MMP-2/-9 probe, the turnover of the broad-range probe was slower at physiological conditions than at lower temperatures, with *k*_cat_/*K*_M_ at 310 K for the broad-range probe reaching only a third its value at 298 K. One explanation may be that at higher temperature, the broad-range probe may occupy a different conformation, making it less readily accepted by MMP-9 and thereby increasing *K*_M_ and/or decreasing *k*_cat_ for the generic probe.

When compared to the FRET substrate described by Knight et al. [6], the NMR probe is substantially more slowly turned over, an observation which can potentially be attributed to the relatively bulky Gd^III^-DOTA chelate reducing the MMP-9 binding affinity for the NMR probe.

### 3.2. Selectivity of the Probe

To test the specificity of the probe, the MMP-2/-9 probe was incubated with MMP-1 and MMP-12, leading to no observable cleavage, as confirmed by mass spectrometry to detect the cleaved products (data not shown). The broad-range probe however responded as expected to all MMPs tested (MMP-1, MMP-2, MMP-9, and MMP-12).

### 3.3. Transverse and Longitudinal ^19^F Relaxation of the 0.1 mM Biosensor in Aqueous Solution

Longitudinal ^19^F relaxation times *T*_1_ for the fluorinated probes and transverse relaxation times *T*_2_^*∗*^, determined by inversion recovery experiments and from the fluorine linewidth (FWHM) [34], respectively, are listed in Table 2. Upon cleavage, the two different MMP probes display drastic changes in their *T*_1_ and *T*_2_^*∗*^ times. The MMP-2/-9 probe shows an increase in *T*_1_ by a factor of over 60, whereas the generic probe increases the *T*_1_ time by a factor of 75. Cleavage also leads to an increase in *T*_2_^*∗*^ by a factor of about 7 and 10 for the MMP-2/-9 probe and the generic probe, respectively, and by a factor of 20 in the previous work at 7 Tesla [25, 26]. The linewidth effect, utilized in previous literature, may therefore lead to a less pronounced change in the MRI contrast than that caused by the reduction of the longitudinal relaxation. Note that however the relaxation differences between the intact and the cleaved sensor are strongly dependent on the sensor concentration as explored in detail further below.

### 3.4. ^19^F Relaxation Behaviour of the Biosensor at 9.4 Tesla

The data in Figure 2 and Table 2 were obtained with a 5 mm high-resolution probe at 14.1 Tesla (564.6 MHz ^19^F resonance frequency) and a biosensor concentration of 0.1 mM. The biosensor concentration was increased to ≥0.4 mM to provide sufficient signal intensity for molecular ^19^F·MRI at 9.4 Tesla (376.4 MHz ^19^F resonance frequency) using a 30 mm ^19^F microimaging coil for excitation and detection. To dissolve the biosensor up to a 1.2 mM concentration, a 9 : 1 (v/v) H_2_O/acetonitrile mixture was used as a solvent at 293 K. The effect of acetonitrile on the kinetics of biosensor cleavage by the enzyme MMP-2 was studied in assays (not shown here). Although the presence of 10% ACN reduced the reaction kinetics, it still allowed for cleavage of the sensor with MMP-2. All relaxation measurements at 9.4 T were performed at a temperature of 293 K.

The biosensor concentration [Rx] dependence of the ^19^F relaxation rate, i.e., the biosensor ^19^F relaxivity, is crucial for the concept development of molecular MRI contrast explored here. As shown in Figure 3 and listed in Table 3, the intact MMP-2/-9 biosensor does not exhibit a concentration dependence for the longitudinal relaxation with *T*_1_ = 10.7 ± 1.0 ms. The observed value is about 30% shorter than the value listed in Table 2 with *T*_1_ = 15.1 ms for the intact biosensor at 0.1. mM concentration obtained in aqueous solution (containing 10% D_2_O) at 14.1 Tesla magnetic field strength and 298 K. Similarly, the transverse relaxation of the intact biosensor in Figure 3 exhibited little concentration dependence, and the average value was found to be *T*_2_ = 3.46 ± 0.42 ms (note that *T*_2_ relaxation times and rates, as determined by spin echo measurements, are reported here as they provide more precise data than *T*_2_^*∗*^ measurements that are affected by sample susceptibility). Upon catalytic cleavage with MMP, the relaxation rates are dramatically reduced. At the same time, the relaxation rates start to exhibit a strong dependence upon (cleaved) biosensor concentration [Rx]. However, even at a relatively high concentration of [Rx] = 1.0 mM, the ^19^F longitudinal relaxation rate is slowed down by a factor of 25 upon cleavage, leading to a longitudinal relaxation time of *T*_1_ = 267 ± 37 ms. Similarly, transverse relaxation is slowed 20-fold upon cleavage, resulting to a transverse relaxation time of *T*_2_ = 72.7 ± 2.5 ms.

The transverse and longitudinal relaxivity (i.e., [Rx] dependence of ^19^F relaxation) obtained from data in Figure 3 is listed in Table 3. The *T*_1_ relaxivity for the cleaved biosensor interpolated for 9 : 1 (v/v) H_2_O/ACN solutions to a concentration of [Rx] = 0.1 mM (Figure 3 and Table 3) is *T*_1_ = 796 ms, a value that falls about 17% short of the value *T*_1_ = 960 ms listed in Table 2 for the aqueous solution (10% D_2_O), indicating a qualitatively very similar relaxation behaviour of the cleaved biosensor within the two solvents.

### 3.5. Molecular Imaging Using Negative *T*_1_ Weighted MRI Contrast through PRE Deactivation

The results of Figure 3 show that, upon cleavage, both transverse and longitudinal relaxations slow down by more than an order of magnitude, even if very high concentrations of up to 1.2 mM biosensor are being used. The effect on transverse relaxation has been exploited in the past to generate positive MRI contrast through increasing ^19^F biosensor signal intensity that indicates the presence of MMP. Figure 4 shows a repeat of this concept with the MMP-2/-9-specific biosensor (Figure 4(c)) but also demonstrates (Figure 4(b)) that *T*_1_ contrast can be exploited through ultrashort imaging time (UTE) ^19^F·MRI [28, 36, 37]. The *T*_1_ weighted MRI produces a negative contrast that is caused by the deactivation of the paramagnetic relaxation effect (PRE) on the ^19^F signal upon cleavage through MMP. An important advantage of the *T*_1_ weighted contrast, as for PRE in general, is the fast data acquisition that significantly reduces the required experimental time.

The UTE images in Figure 4 were employed to allow for MRI of the intact biosensor despite the short *T*_2_ times and fast repetition times (5 ms recycle delay) enabled recording within 6 min each, whereas the spin echo MR images that used *T*_2_ contrast took 75 min each due to the long recycle delay of 200 ms to allow for sufficient, but still incomplete, *T*_1_ relaxation of the cleaved sensor. *T*_1_ weighted ^19^F·MRI contrast of the intact biosensor with active PRE benefits from the UTE methodology that enables the recording of signals with very short *T*_2_ times. However, as shown in Table 3, the intact biosensor has a fairly large transverse to longitudinal relaxation ratio of *T*_2_/*T*_1_≈ 0.3 with *T*_2_ = 3.46 ms and UTE MRI, although helpful, is not absolutely required. UTE MRI is an excellent methodology for systems with very short transverse relaxation times, but it is experimentally demanding (i.e., requires trajectory calibration), in particular if used for systems with inherently low signal intensity. Figure 5 demonstrates that, for the particular molecule used in this study, ^19^F biosensor imaging is also feasible through a simple gradient echo (GE) sequence. The ^19^F·GE·MR images shown in Figures 5(b) and 5(d) were each recorded within 16 min using an echo time of TE = 0.873 *μ*s (the shortest possible with the hardware used), signal averages NS = 1536, and a repetition time of TR = 20 ms. Figure 5(b) shows three sample tubes with 0.6 mM biosensor solution. The biosensor in all three sample tubes was 0.6 mM (i.e., half the concentration of the samples used for UTE MRI in Figure 4).

The appearance of negative ^19^F contrast of the biosensor obtained through the *T*_1_ weighted protocols described in this work indicates the presence of MMP through responsive PRE deactivation. However, transport phenomena within *in vivo* organisms may cause negative contrast through biosensor dilution and therefore would produce a false-positive response. To distinguish the MMP catalysed biosensor reaction from the false positive due to biosensor dilution, it is instructive to utilize additional *T*_1_ sensitive ^1^H·MRI measurements. Figure 5(b) shows the ^19^F·MRI of three sample tubes, (i), (ii), and (iii) that contain the intact biosensor at 0.6 mM concentration. In Figure 5(d), only sample tube (i) contains the intact biosensor at this concentration while MMP-2 was administered to sample (ii), and sample tube (iii) was diluted to 0.4 mM concentration of the intact biosensor. The ^19^F·MR image in Figure 5(d) shows very similar reduction in intensity for the two samples (ii) and (iii). After >5 h incubation with MMP, the ^19^F longitudinal relaxation time of sample (ii) displayed an increase from *T*_1_ = 10.7 ms for the intact sensor to approximately *T*_1_ = 275 ± 15 ms (i.e. using single exponential fit), a value that remained stable throughout the further experiments. The observed ^19^F relaxation time value falls a little short of *T*_1_ = 380 ms, the expected value from Table 3 for the completely reacted sensor at 0.6 mM concentration. The cause of this deviation was not further investigated as it is of little consequence for the proof of concept demonstrated here. For better visualization, Figure 5(f) shows the result from the subtraction of the MR image of Figure 5(d) from Figure 5(b).

The *T*_1_ relaxation in ^1^H·MRI is strongly affected by the paramagnetic Gd(III) concentration, but the relaxation is always intermolecular in nature. Therefore, unlike ^19^F·*T*_1_ relaxation, the ^1^H·*T*_1_ relaxation is not affected by the cleavage of the gadolinium group from the ^19^F containing moiety. The ^1^H longitudinal relaxation of the 0.6 mM intact sensor in (ii) was found to be *T*_1_ = 320 ms both before and after incubation with MMP. Figure 5(a) shows the ^1^H gradient echo MRI of three tubes with 0.6 mM biosensor before MMP and dilution; tube (vi) contains 0.4 mM of a completely cleaved biosensor (recycled from a previous experiment) that causes a weak signal in Figures 5(b) and 5(d); and sample tube (v) contains only PBS solution without ^19^F or gadolinium. Figure 5(c) depicts ^1^H null point MRI using inversion recovery protocol with an inversion recovery time TI = 220 ms that causes all signals from tubes containing 0.6 mM biosensor solution to vanish due to zero crossing.

Figure 5(e) shows the result from an identical ^1^H null point MRI protocol but with the samples as in Figure 5(d) (i.e., (ii) partially reacted through MMP and (iii) diluted to 0.4 mM intact biosensor). As expected, there is no observable change in ^1^H·*T*_1_ relaxation of the reacted biosensor in (ii), but the diluted sample in (iii) leads to a strong inversion recovery signal increase due to reduced ^1^H·*T*_1_ relaxation that produces a clearly visible ^1^H·MRI response. The ^1^H·MRI response, shown in Figure 5(e), identifies the ^19^F·MRI response for sample (iii) as false-positive. For better visualization, Figure 5(g) depicts the ^1^H null point MRI difference between Figures 5(e) and 5(c).

### 3.6. Towards In Vivo MRI: Tasks for MMP Biosensor Development

The results presented in Figures 4 and 5 demonstrate the effectiveness of responsive PRE contrast for molecular imaging but also indicate where future development is required. False-positive signals due to biosensor dilution can be eliminated by fast null point ^1^H·MRI measurements of H_2_O that was completed within a total recording time of 2½ min in Figure 5. Within an organism, the biosensor localization may not be known quantitatively and, additionally, dilution due to transport mechanisms will occur over time. Therefore, ^1^H·MRI·*T*_1_ maps may provide detailed insights into biosensor concentration throughout the organism. To generate “snapshots” of the concentration distribution, the *T*_1_ maps need to be recorded quickly which should not be a problem as abundant water molecules are being detected and improved ^1^H·MRI protocols and/or reduced resolution may accelerate the acquisition further. Detailed Gd^III^ concentration maps will then enable ^19^F·PRE·MRI data analysis to extract quantitatively the extent of the reaction. It is important to note that the previously explored transverse (*T*_2_^*∗*^) relaxation-based biosensor detection concept can also benefit from ^1^H·MRI·*T*_1_ maps. In analogy to a false-positive response with a PRE-based biosensor, a false-negative response may occur for *T*_2_^*∗*^ biosensors because dilution will diminish the ^19^F·MRI signal, and this can mask the presence of the target enzyme. As a word of caution for NMR spectroscopic studies, the rate of enzymatic hydrolysis of the probes was found to be significantly affected by the percentage of D_2_O present in the solution.

To advance the responsive PRE deactivation concept towards *in vivo* applications, future biosensor synthesis will need to address three key issues: (1) The biosensors will need to be fully water soluble to enable the concentration required for MRI. At the same time, the water soluble biosensor needs to be stable within the *in vivo* environment. (2) A relatively high concentration of the probe molecule was required in order to perform the MR imaging experiment in the current and previous studies. The ^19^F signal intensity [29] requires further improvement to be viable for *in vivo* applications. Generally, various successful efforts have been made to improve the signal intensity by increasing the ^19^F spins per molecule unit [38, 39], and these approaches need to be explored to make responsive PRE deactivation more biocompatible by reducing the required concentration of biosensor molecules. Furthermore, reducing the required biosensor concentration will also enhance the molecular MRI contrast due to an increased *T*_1_ relaxivity gap between the PRE-activated and the PRE-deactivated probe molecule. As shown in Figure 3, the relaxivity of the “off-state” exhibits a strong concentration dependence while the “on-state” relaxivity is largely unaffected by concentration changes. (3) A paramagnetic group producing fast *T*1 but relatively slow *T*2 relaxation (i.e., a high ratio close to the limit *T*2/*T*1 = 1) will further improve the presented concept. The paramagnetic system used in this work, Gd^III^, generally produces very unfavourable *T*_2_/*T*_1_ ratios that are also dependent on the separation between the paramagnetic centre and the fluorine spins [40]. Distance cannot always be freely selected in functionalized sensors but a variety of better options than Gd^III^ for the paramagnetic centre, such as Fe^II^, Tm^III^, and Ho^III^, have been explored to improve ^19^F·MR signal intensities through increased the *T*_2_/*T*_1_ ratios [34, 39, 40]. Combining high *T*_2_/*T*_1_ ratios with an increased number of ^19^F spins, Kislukhin and coworkers have reported promising results with paramagnetic fluorinated nanoemulsions [28].

Lastly, the presented concept may also work for molecular imaging with hyperpolarized (hp) ^129^Xe, a contrast agent that is becoming more readily available [41, 42]. A recent study demonstrated a conceptual “on/off” probe with a cryptophane cage that temporarily binds xenon atoms and brings them into close proximity to the paramagnetic Gd^III^ centre. Deactivation of the paramagnetic relaxation of the probe molecule caused an eight-fold reduction in the *T*_1_ relaxation of ^129^Xe in the solvent [43]. Note that hp ^129^Xe can in principle be added long after the probe molecule has been administered to an organism and after any deactivating reaction has occurred in regions with up-regulated biomarker targets. Replacement of ^19^F with exogenous hp ^129^Xe for MMP biosensor probe molecules may therefore be an option to significantly increase the signal from such enzymatic probes.

## 4. Conclusions

The usage of responsive PRE deactivation for *T*_1_ weighted ^19^F·MRI contrast, presented in this work, enables accelerated observation of MMP enzyme activity by taking advantage of the significantly reduced imaging time compared to the previously utilized *T*_2_ or *T*_2_^*∗*^ weighted MRI. UTE ^19^F·MRI can be employed if problems associated with short ^19^F·*T*_2_ relaxation need to be overcome. In this work, UTE·^19^F·MRI allowed for a more than 12-fold reduction in the total acquisition time compared to a *T*_2_ weighted spin echo MRI protocol. Alternatively, even a simple *T*_1_ weighted gradient echo sequence enabled molecular MRI of MMP-2 within a short acquisition time and with a reasonable signal to noise ratio despite the unfavourable *T*_2_/*T*_1_ ratio of the Gd^III^ system used in this proof of concept study. Responsive PRE deactivation for *T*_1_ weighted MRI leads to a negative contrast where the intact biosensor is observed and the disappearance of the MRI signal indicates cleavage due to enzyme activity. False-positive identification, where vanishing ^19^F·MRI signals are caused by biosensor dilution, can be identified through additional *T*_1_ weighted ^1^H·MRI that is not affected by the biosensor cleavage. More progress is needed to advance targeted biosensors with responsive PRE deactivation towards *in vivo* and clinical applications. This includes improved molecular design of ^19^F biosensors but also the advancement of hyperpolarized ^129^Xe biosensors with responsive deactivation of paramagnetic relaxation [43]. If successful, this concept offer significant prospects for monitoring disease progression and treatment impact with much improved precision and therefore could play an important role in personalized medicine.

## Figures and Tables

**Table 1:** *k*_cat_/*K*_M_ for both probes under all conditions.

Probe	Solvent	Temp (K)	*k*_cat_/*K*_M_ (M^−1^·s^−1^)
MMP-2/-9	H_2_O	310	726
MMP-2/-9	H_2_O	298	267
MMP-2/-9	D_2_O	298	56.5
Generic	H_2_O	310	75.9
Generic	H_2_O	298	207
Generic	D_2_O	298	108

H_2_O as a solvent denotes v/v 9 : 1 H_2_O/D_2_O.

**Table 2:** Relaxation times for MMP probes (0.1 mM) with MMP-2/-9 and generic sequence specificity at 14.1 T and at 298 K using v/v 9 : 1 H_2_O/D_2_O as a solvent.

	Cleavage state	*T*_1_ (ms)	*T*_2_^*∗*^ (ms)^a^
MMP-2/-9-specific sequence	Pre	15.1	<2.6^b^
	Post	961	18.2
Generic MMP sequence	Pre	12.8	<2.3
	Post	960	23.8

^a^The *T*_2_^*∗*^ value was calculated using the relation, *T*_2_^*∗*^  =(*π*Δ*v*)^−1^, where Δ*v* is the linewidth of the fluorine peak at full width half maximum. ^b^The inability to properly identify the exact linewidth of the precleaved state due to the broad linewidth; hence, the maximum linewidth is used, yielding a minimum *T*_2_^*∗*^ value.

**Table 3:** Biosensor relaxivity for ^19^F at 293 K and 9.4 Tesla obtained from Figure 3 relaxation data fitting using equations 1/*T*_1_ = *ρ*_1_ = *ρ*_1_^[Rx]^ ∗ [Rx] + *ρ*_1_^0^ and 1/*T*_2_ = *ρ*_2_ = *ρ*_2_^[Rx]^ ∗ [Rx] + *ρ*_2_^0^.

	Intact biosensor	Cleaved biosensor
*ρ*_1_	*ρ*_1_ = 92.9 ± 7.3 s^−1^	*ρ*_1_^[Rx]^ = 2.76 ± 0.40 s^−1^ mM^−1^
	*T*_1_ = 10.7 ± 1.0 ms (Figure 3(a))	*ρ*_1_^0^ = +0.98 ± 0.33 s^−1^ (Figure 3(c))
*ρ*_2_	*ρ*_2_ = 289 ± 4 s^−1^	*ρ*_2_^[Rx]^ = 9.85 ± 0.37 s^−1^·mM^−1^
	*T*_2_ = 3.46 ± 0.42 ms (Figure 3(b))	*ρ*_2_^0^ = 3.90 ± 0.30 s^−1^ (Figure 3(d))

Data are for biosensor with MMP-2/-9-specific sequence using MMP-2 as the cleavage enzyme.

## Data Availability

The chemical synthesis data used to support the findings of this study are included in the supplementary information. The NMR spectroscopic and MRI data used to support the findings of this study are available from the corresponding author upon request.

## Supplementary Materials

Supplementary Materials Scheme S1: synthesis of fluorinated amino acid (4). Scheme S2: synthesis of “clickable” GdIII chelate. Scheme S3: SPPS of the broad-range probe and attachment of the GdIII-DOTA chelator (1).
