# Supplementary material for: Accelerated 19F·MRI Detection of Matrix Metalloproteinase-2/-9 through Responsive Deactivation of Paramagnetic Relaxation Enhancement
Source: Contrast Media Mol Imaging. 2019 Feb 28;2019:4826520. doi: 10.1155/2019/4826520 (PMC6421815; doi:10.1155/2019/4826520)
Supplement: Supplementary Materials — Scheme S1: synthesis of fluorinated amino acid (4). Scheme S2: synthesis of “clickable” GdIII chelate. Scheme S3: SPPS of the broad-range probe and attachment of the GdIII-DOTA chelator (1). [file 4826520.f1.pdf]

Contrast Media & Molecular Imaging

Supplementary Materials for:

Accelerated ^{19}F MRI detection of matrix metalloproteinase-2/-9 through responsive deactivation of paramagnetic relaxation enhancement.

Henryk M. Faas[1,2,*], James L. Krupa[3], Alexander J. Taylor[1,2,£], Francesco Zamberlan[3,#], Christopher J. Philp[1,4], Huw E. L. Williams[3], Simon R. Johnson[4], Galina E. Pavlovskaya[1,4], Neil R. Thomas[3,*] , Thomas Meersmann[1,4,5,*]

[1]Sir Peter Mansfield Imaging Centre, University of Nottingham, Nottingham, NG7 2RD, United Kingdom.
[2]Division of Clinical Neuroscience, School of Medicine, University of Nottingham, Nottingham, NG7 2UH, United Kingdom.
[3]Centre for Biomolecular Sciences, School of Chemistry, University of Nottingham, Nottingham, NG7 2RD, United Kingdom.
[4]Respiratory Medicine and Biomedical Research Centre, School of Medicine, University of Nottingham, Nottingham, NG7 2UH, United Kingdom.
[5]Department of Electrical and Electronic Engineering, University of Nottingham, Ningbo, PR China.

[£]Current address: Medical Physics, Nottingham University Hospitals NHS Trust, Nottingham, NG7 2UH, United Kingdom

[#]Current address: School of Chemistry, University of Lincoln, Brayford Pool, Lincoln, LN6 7TS, United Kingdom.

*Correspondence should be addressed to:
Thomas Meersmann, Thomas.Meersmann@nottingham.ac.uk; Henryk M. Faas, Henryk.Faas@nottingham.ac.uk; Neil R. Thomas, Neil.Thomas@nottingham.ac.uk

S1: Synthesis overview

The synthesis began with 3,5-*bis*(trifluoromethyl) benzylamine (**2**), an inexpensive reagent which provided six magnetically and chemically equivalent fluorine atoms. This benzyl amine was then coupled to Fmoc-Asp-(tBu)-OH using *N*-(3-dimethylaminopropyl)-*N'*-ethylcarbodiimide hydrochloride (EDC), yielding (**3**). Following the coupling, the *tert*-butyl ester was hydrolysed under acidic conditions resulting in the formation of the free acid (**4**) as shown in scheme S1. This was then loaded onto at 2-chlorotrityl chloride resin for use in solid phase peptide synthesis (SPPS).

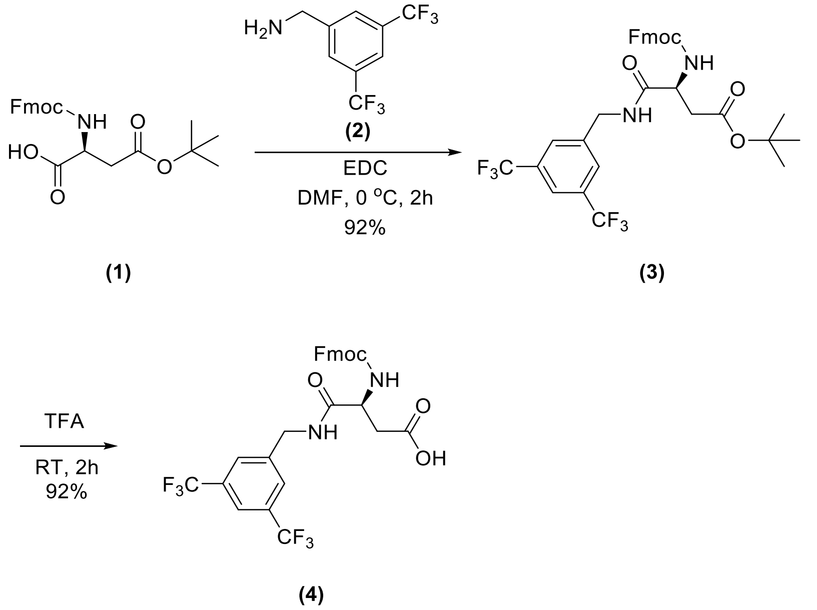

Scheme S1: Synthesis of fluorinated amino acid (**4**).

Synthesis of an alkyne-derivatized Gd^{III}-DOTA (**11**) began with the selective *N*-alkylation of cyclen (**7**), the parent ring of DOTA, using three stoichiometric equivalents of *tert*-butyl bromoacetate to furnish the tri-alkylated DO3A product (**8**) in excellent yield (scheme S2). This consistently reliable step gave reproducible high yields provided the concentration of the reagents was carefully controlled; the reaction was observed to achieve the highest yields of the desired product with 1 mL of solvent per 1 mg of cyclen. The following step was the alkylation of the remaining secondary amine of DO3A-derivative (**8**),

and this was achieved using *N*-propargyl-α-bromocarboxamide (**6**). Alkyne (**6**) was synthesised in reasonable yield by the acylation of propargylamine using bromoacetyl bromide. Alkyne (**6**) was then used to alkylate DO3A (**8**), giving the main scaffold. The *tert*-butyl esters of the DOTA-alkyne (**9**) were subsequently removed under acidic conditions with formic acid, resulting in the deprotected chelate (**10**). The unprotected chelate (**10**) was then used to coordinate to Gd^{III} from $GdCl_3$ in aqueous solution at pH 7 to give the desired product (**11**) in excellent yield.

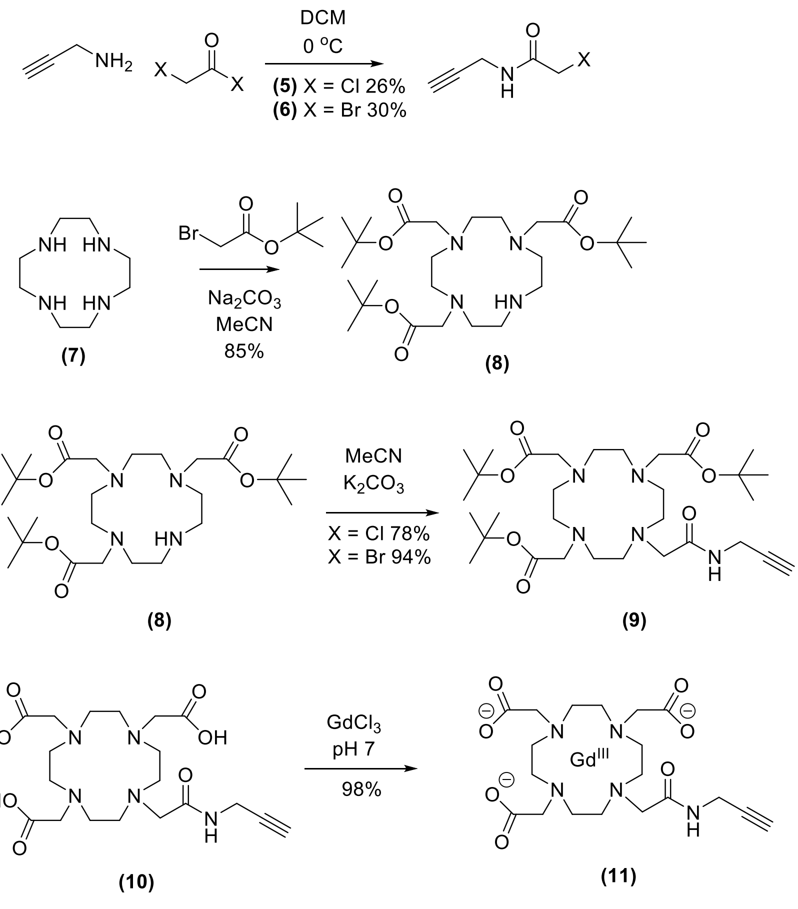

Scheme S2: Synthesis of "clickable" Gd^{III} chelate.

The peptide sequences used were based on previously published work for substrates found to be selective to the indicated MMPs. We chose to use a broad range MMP sequence, PLGLWAR, which has been reported to be rapidly cleaved by multiple different MMPs. In

addition, we also selected a sequence shown to be selective for MMP-2 and MMP-9 only, PLGMWSR [1, 2].

Using SPPS we were then able to build the desired peptide sequence, using 20% piperidine in DMF and PyOxim as Fmoc deprotection and coupling agents respectively, to extend the peptide as desired (see scheme S3). The difficult step within the synthesis was the coupling of Gd^{III} chelate onto the terminal proline. Initial attempts to couple DOTA-*tris* (tri-*tert*-butyl ester, mono acid) onto the terminal proline of the peptide sequence proved to be ineffective in our hands, subsequent attempts with various linkers and analogues of DOTA-based compounds also proved inefficient (not discussed here). In order to overcome this step, we used 'click' chemistry as an alternative to amide coupling agents. Azide labelling of the peptide was achieved by synthesising 3-azidopropanoic acid (**12**), in good yield from 3-bromopropanoic acid, and this was then coupled onto the peptide using PyOxim. Utilising Cu^{I}-catalysed 'click' chemistry meant that pre-loading the DOTA with Gd^{III} was essential as non-loaded DOTA allowed Cu ion chelation to occur. The 'click' reaction proved to be effective both whilst the peptide was still bound to the resin and post-cleavage from the resin (scheme S3), giving overall yields of 3% and 4% for the generic and MMP-2/-9 probes respectively.

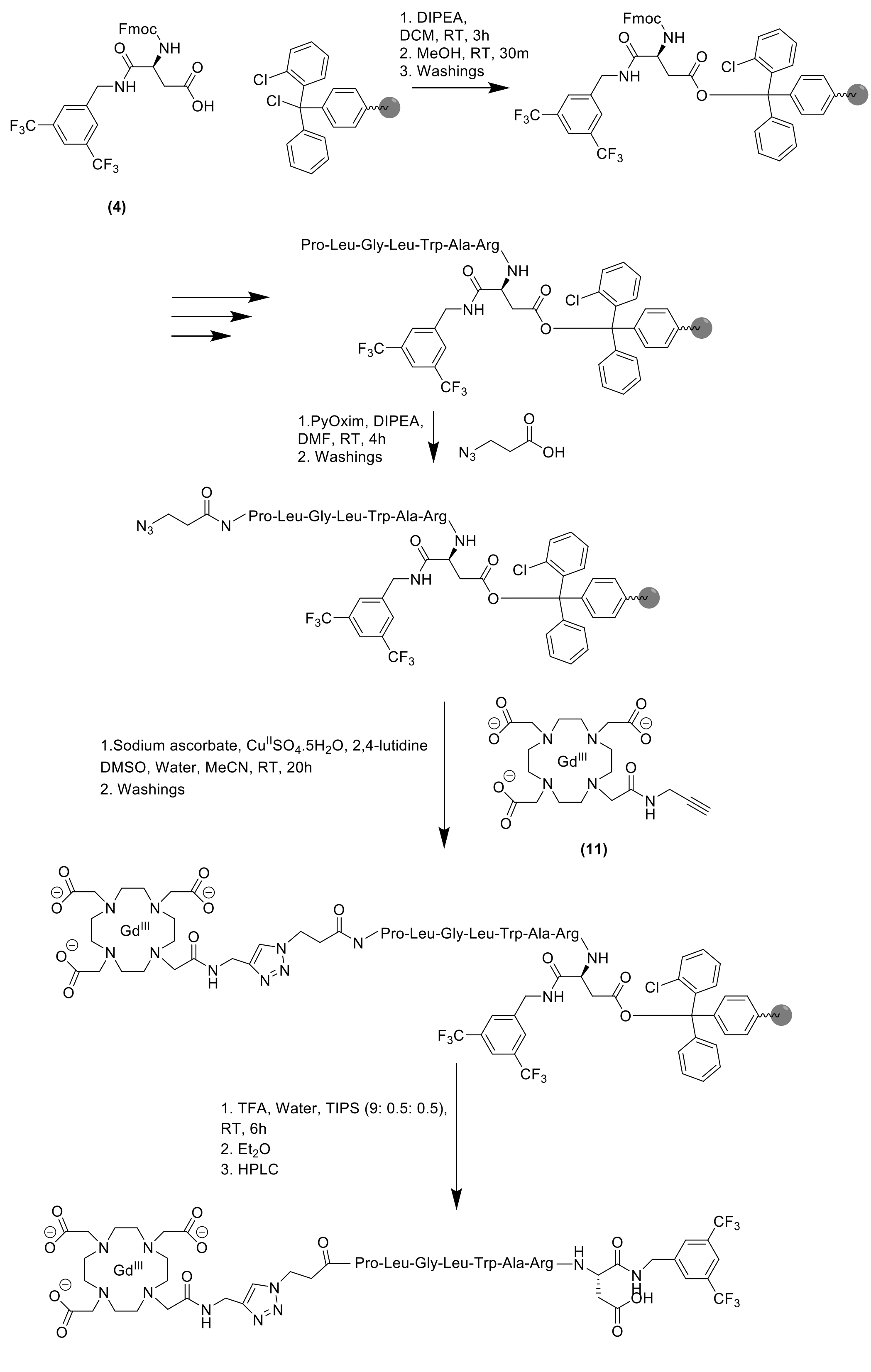

Scheme S3: SPPS of the broad-range probe and attachment of the Gd^{III}-DOTA chelator (**1**)

S2: Synthesis details

DMF (peptide synthesis grade) was supplied by Rathburn Chemicals Ltd, anhydrous DMF was purchased from Sigma Aldrich®; all reagents were purchased from either Sigma Aldrich®, Alfa Aesar®, Merck Chemicals Ltd, or Strem Chemicals Inc; HPLC-grade solvents purchased from Fisher Scientific® were used for all reactions

Chemical shifts (δ) are given in parts per million (ppm) and *J* values in Hertz (Hz). Multiplets are designated by the following notations: singlet (s), doublet (d), triplet (t), quartet (q), multiplet (m). All ^{1}H NMR spectra were recorded on Bruker™ AV400, AV(III)400, or DPX 400 spectrometers at 400 MHz, or AV(III)500 at 500 MHz and at ambient temperature. All spectra were recorded relative to residual solvent peaks. Spectra were recorded in solutions of deuterated chloroform ($CDCl_3$, δ_{solv} = 7.26), deuterated methanol (CD_3OD, δ_{solv} = 3.31), deuterated water (D_2O, δ_{solv} = 4.79), or deuterated DMSO ($(CD_3)_2SO$, δ_{solv} = 2.50) [3]. All ^{13}C NMR spectra were recorded on Bruker™ AV400, AV(III)400, or DPX 400 spectrometers at 100 MHz, or AV(III)500 at 125 MHz and at ambient temperature. All spectra were recorded relative to residual solvent peaks. Spectra were recorded in solutions of deuterated chloroform ($CDCl_3$, δ_{solv} = 77.1), deuterated methanol (CD_3OD, δ_{solv} = 49.0), deuterated water (D_2O, δ_{solv} = no signal), or deuterated DMSO ($(CD_3)_2SO$, δ_{solv} = 39.5) [3]. All ^{19}F NMR spectra were recorded on a Bruker™ AV(III)400, or DPX 400 spectrometer at 376 MHz and at ambient temperature. Assignments were based on DEPT 90, DEPT 135, and HMQC spectra. High field NMR work was performed on a AV(III)600 MHz. High field ^{19}F NMR was performed solely on the AV(III)600 MHz which was fitted with an fluorine probe.

High resolution mass spectroscopy (HRMS) was recorded on a Bruker™ microTOF, an orthogonal Time of Flight instrument with electrospray ionisation (ESI, both positive and negative ion) sources as indicated. Values of mass to charge ratio (m/z), are given to four decimal places. The mass of the counter ions are H^+ 1.0078, and Na^+ 22.9898.

Infared spectroscopy was recorded on either a Thermo Scientific NICOLET IR200 FT-IR infared spectrometer, with samples prepared as a nujol mull on NaCl discs or as KBr discs.

Solution IR was recorded on a Bruker Tensor 27 FT-IR instrument using spectroscopic grade chloroform or methanol.

UV spectroscopy to monitor amino acid resin loadings was measured on either a Cary 100 Bio UV-visible spectrophotometer, or a WPA lightwave set to 290 nm.

Thin layer chromatography was performed using Merck Kieselgel 60 F_{254} plates. Visualisation was by UV light and staining with phosphomolybdic acid (PMA) or ninhydrin with heating. Flash column chromatography was performed using Merck Kieselgel silica gel 60 Å, 230-400 mesh, 40-63μm, unless otherwise stated.

Melting points were determined on a Stuart Scientific melting point apparatus (SMP3), values are given in degrees Celsius (˚C) and are uncorrected.

Solid phase peptide synthesis was performed using a NovaSyn®gem Peptide Synthesiser set to a flow rate of 3 mL/min, attached to a Gilson 115 UV detector to monitor Fmoc absorbance at 290 nm. All resins were loaded into Omnifit Ltd Solventplus 10 mm x 100 mm columns.

All HPLC were run on an Agilent 1200 series system. Analytical HPLC was performed using an Agilent Eclipse XDB-C18 analytical column 4.6 x 150 mm, with a 5 μm pore size. Semi-preparative HPLC was performed using either an Agilent Eclipse XDB-C18 semi-preparative column 9.4 x 150 mm, with a 5 μm pore size, or an Agilent Eclipse XDB-C18 semi-preparative column 9.4 x 250 mm, with a 5 μm pore size. Solvent A was 0.1% formic acid in milli Q water, and solvent B was 0.1% formic acid in HPLC grade acetonitrile.

2-Bromo-N-(prop-2-yn-1-yl)acetamide (**6**): Bromoacetyl bromide (460 mg, 2.28 mmol, 1.1 eq.) and potassium carbonate (590 mg, 4.28 mmol, 2.0 eq.) were stirred under a nitrogen atmosphere at 0 ˚C in anhydrous dichloromethane (5 mL). Propargyl amine (120 mg, 2.18 mmol, 1.0 eq.) in anhydrous dichloromethane (1 mL) was slowly added dropwise over 30 minutes, and stirred for two hours at ambient temperature before being quenched with water (2 mL), followed by a 24 hour stir at ambient temperature. The product was then diluted with dichloromethane (30 mL) and washed with citric acid (5% w/v solution, 2 x 10 mL) and water

(2 x 10 mL). The organic phase was then dried over anhydrous sodium sulfate, filtered and concentrated *in vacuo* to yield the desired product (110 mg, 30%) as an off white solid. Mp. 66-67 ˚C; IR V_{max} (Nujol)/cm^{-1} 3291 (NH), 1642 (C=O); ^{1}H NMR (400 MHz, $CDCl_3$) δ 2.30 (t, *J* = 4.0 Hz, 1H, alkyne-H), 3.90 (s, 2H, CH_2), 4.09 (dd, *J* = 5.4, 2.6 Hz, 2H, CH_2), 6.94 (bs, 1H, N*H*). ^{13}C NMR (100 MHz, $CDCl_3$) δ 28.6 (*C*H_2-Br), 29.9 (*C*H_2NH), 72.2 (*C*q alkyne), 78.6 (*C*H), 165.5 (*C*=O). HRMS (ESI) required for $C_5H_6BrNONa^+$ 197.9525 observed MNa^+ 197.9552. HRMS (ESI) required for $C_5H_6BrNONa^+$ 199.9505 observed MNa^+ 199.9525.

2-Chloro-N-(prop-2-yn-1-yl)acetamide (**5**): Chloroacetyl chloride (260 mg, 2.30 mmol, 1.1 eq.) and potassium carbonate (590 mg, 4.28 mmol, 2.0 eq.) were stirred under a nitrogen atmosphere at 0 ˚C in anhydrous dichloromethane (5 mL). Propargyl amine (120 mg, 2.18 mmol, 1.0 eq.) in anhydrous dichloromethane (1 mL) was slowly added dropwise over 30 minutes, and stirred for two hour at ambient temperature before being quenched with water (2 mL), followed by a 24 hour stir at ambient temperature. The product was then diluted with dichloromethane (30 mL) and washed with citric acid (5% w/v solution, 2 x 10 mL) and water (2 x 10 mL).The organic phase was then dried over anhydrous sodium sulfate, filtered and concentrated *in* vacuo to yield the desired product (70 mg, 26%) as an off white solid. Mp. 67-68 ˚C. IR V_{max} (Nujol)/cm^{-1} 3291 (NH), 1642 (C=O); ^{1}H NMR (400 MHz, $CDCl_3$) δ 2.30 (t, *J* = 2.6 Hz, 1H, alkyne-H), 4.08 (s, 2H, CH_2), 4.10 (dd, *J* = 5.4, 2.5 Hz, 2H, CH_2), 6.93 (bs, 1H, N*H*). ^{13}C NMR (100 MHz, $CDCl_3$) δ 29.6 (*C*H_2NH), 42.4 (*C*H_2-Cl), 72.1 (*C*q alkyne), 78.7 (*C*H), 165.8 (*C*=O). HMRS (ESI) cal. MW of 135 is below the lower detection limit (150 amu) of instrument.

Tri-tert-butyl 2,2',2''-(1,4,7,10-tetraazacyclododecane -1,4,7-triyl)triacetate (**8**): To a solution of cyclen (200 mg, 1.16 mmol, 1.0 eq.) in acetonitrile (150 mL), sodium carbonate (350 mg, 3.30 mmol, 3.0 eq.) was added to give a white suspension that was stirred for 15 minutes under a nitrogen atmosphere. A solution of *tert*-butyl bromoacetate (660 mg, 3.40 mmol, 3.0 eq.) in acetonitrile (50 mL) was slowly added dropwise over 15 minutes and the resulting mixture stirred for 30 minutes at ambient temperature, followed by an 18 hour reflux

at 80 ˚C. The white precipitate was then filtered and recrystallised from the minimum amount of boiling toluene and washed with ice cold diethyl ether (20 mL) to yield the desired product (590 mg, 85%) as a fine white powder. Mp. 192-193 °C (Lit. 190-191 °C); IR V_{max} (KBr)/cm^{-1} 3428 (N-H), 1712 (C=O); ^{1}H NMR (400 MHz, $CDCl_3$) δ 1.46 (s, 27H, 3 x -C(CH_3)$_3$), 2.85-2.95 (m, 12H, 6 x CH_2), 3.11 (s, 4H, 2 x CH_2), 3.29 (s, 2H, CH_2), 3.37 (s, 4H, 2 x CH_2). ^{13}C NMR (100 MHz, $CDCl_3$) δ 28.2 (-C(*C*H_3)$_3$), 28.3 (-C(*C*H_3)$_3$), 47.6 (*C*H_2), 49.1 (*C*H_2), 51.1 (*C*H_2), 58.2 (*C*H_2), 81.7 (2 x -*C*(CH_3)$_3$), 81.9 (-*C*(CH_3)$_3$), 169.6 (*C*=O), 170.5 (2 x *C*=O). HRMS (ESI) required for $C_{26}H_{51}N_4O_6^+$ 515.3803 observed MH^+ 515.3819.

Tri-tert-butyl2,2',2''-(10-(2-oxo-2-(prop-2-yn-1-ylamino)ethyl)-1,4,7,10-tetraazacyclododecane-1,4,7-triyl)triacetate (**9**): Cyclen (**8**) (210 mg, 0.490 mmol, 1.0 eq.) and alpha-bromo alkyne (**6**) (80 mg, 0.44 mmol, 1.1 eq.) were dissolved in acetonitrile (10 mL). Potassium carbonate (120 mg, 0.870 mmol, 2.0 eq.) was then added. The solution was then stirred for 24 hours at 60 ˚C, and concentrated *in vacuo*. The residue was then dissolved in dichloromethane (10 mL) and successively washed with water (3 x 10 mL). The organic phase was dried over anhydrous sodium sulfate, filtered and concentrated in vacuo to give the desired product (250 mg, 94%) as an off white solid. Mp. 94-95 ˚C; IR V_{max} (Nujol)/cm^{-1} 1721 (C=O), 1680 (C=O); ^{1}H NMR (400 MHz, $CDCl_3$) δ 1.39 (s, 27H, C(CH_3)$_3$), 2.15 (s, 1H, CH_2CC*H*), 2.16-3.94 (m, 24H, NCH_2CH_2N, and CH_2), 3.98 (m, 2H, CH_2CCH), 8.80 (t, *J* = 5.5 Hz, 1H, N*H*); ^{13}C NMR (100 MHz, $CDCl_3$) δ 27.8 (C(*C*H_3)$_3$), 27.9 (C(*C*H_3)$_3$), 28.0 (C(*C*H_3)$_3$), 28.5 (*C*H_2), 29.3 (*C*H_2), 29.4 (*C*H_2), 55.6 (*C*H_2), 56.1 (*C*H_2), 56.2 (N*C*H2*C*H2N, *C*H2), 69.6 (Alkyne *C*H), 71.2 (Alkyne Cq), 80.8 (*C*(CH_3)$_3$), 81.8 (*C*(CH_3)$_3$), 82.0 (*C*(CH_3)$_3$), 171.8 (C=O), 172.4 (*C*=O), 172.8 (C=O). HRMS (ESI) required for $C_{31}H_{55}N_5O_7^+$ 610.4179 observed MH^+ 610.4174.

2,2',2''-(10-(2-Oxo-2-(prop-2-yn-1-ylamino)ethyl)-1,4,7,10-tetraazacyclododecane-1,4,7-triyl)triacetic acid (**10**): Protected Alkyne (**9**) (383 mg, mmol, 1 eq.) was dissolved in formic acid (10 mL) and water (0.5 mL) and heated to 60 ˚C for 18 hours under nitrogen. Once at ambient temperature the solvent was removed in vacuo to yield the desired product as

an off white solid (261 mg, 94%). Decomp 224 ˚C; ^{1}H NMR (400 MHz, D_2O) δ 2.53 (apparent q, J = 4.0 Hz, alkyne CH), 2.77-3.99 (m, 26H, NCH_2CH_2N, and CH_2); ^{13}C NMR (100 MHz, D_2O) δ 28.8 (CH_2), 28.9 (CH_2), 29.0 (CH_2), 48.3 (CH_2) 48.6 (CH_2), 50.5 (CH_2), 51.3 (CH_2), 53.4 (CH_2), 55.2 (CH_2), 55.6 (CH_2), 56.1 (CH_2), 69.1 (CH_2), 71.1 (alkyne *C*H), 73.4 (alkyne C_q), 170.1 (*C*=O), 174.3 (*C*=O); HRMS (ESI) required for $C_{19}H_{32}N_5O_7^+$ 442.2296 observed MH$^+$ 442.2294. HRMS (ESI) required for $C_{19}H_{30}N_5O_7^-$ 440.2145 observed MH$^-$ 440.2148.

Gadolinium 2,2',2"-(10-(2-oxo-2-(prop-2-yn-1-ylamino)ethyl)-1,4,7,10-tetraazacyclododecane-1,4,7-triyl) triacetate (**11**): Gadolinium (III) chloride hexahydrate in water (3 mL), was added to chelate (**10**) (277 mg, mmol, 1 eq.) dissolved in water (10 mL). The pH was then adjusted to pH 5 using aqueous potassium hydroxide (0.2 M), after 1 hour the pH was readjusted to pH 5, again using aqueous potassium hydroxide (0.2 M), followed by an 18 hour stir. The solution was then concentrated in vacuo; once dry, the white solid was re-dissolved in ethanol (10 mL), stirred for 30 minutes and filtered, finally concentrated in vacuo to yield the desired product (370 mg, 98%). HRMS (ESI) required for $C_{19}H_{28}GdN_5O_7^+$ 593.1491, 594.1509, 595.1504, 596.1522, 597.1524, 599.1553 observed MH$^+$ 593.1345, 594.1294, 595.1276, 596.1288, 597.1291, 599.1309. HRMS (ESI) required for $C_{19}H_{28}ClGdN_5O_7^-$ 631.0918 observed MH$^-$ 627.0892, 628.0891, 630.0910, 631.0908, 632.0940, 633.0948, 635.0925, 635.0913.

3-Azidopropanoic acid: 3-Bromopropanoic acid (1.93 g, mmol, 1 eq.) was dissolved in dimethylformamide (30 mL), and water (10 mL). To this stirred solution sodium azide (0.87 g, mmol, 1.1 eq.) was then slowly added portionwise over 10 minutes. The solution was heated to 55 ˚C for 18 hours, followed by 6 hours at 67 ˚C. Once at ambient temperature, water (150 mL) and ethyl acetate (20 mL) were then added and the aqueous phase successively extracted with ethyl acetate (2 x 20 mL), and diethyl ether (2 x 20 mL). The organic phases were then combined and dried over magnesium sulfate, filtered and concentrated in vacuo with the water bath at 30 ˚C, to yield the desired product as a free

flowing light yellow liquid (1.45 g, 69 %), which was then re-dissolved in ethyl acetate (5 mL) for storage. IR V_{max} (Nujol)/cm^{-1} 3454 (O-H), 2103 (N_3), 1727 (C=O), 1255 (C-O); ^{1}H NMR (400 MHz, $CDCl_3$) δ 2.66 (t, J = 6.5 Hz, 2H), 3.61 (t, J = 6.5 Hz, 2H), 10.17 (bs, 1H); ^{13}C NMR (100 MHz, $CDCl_3$) δ 33.7 (CH_2), 46.4 (CH_2), 176.4 (C=O).

(S)-Tert-butyl 3-((((9H-fluoren-9-yl) methoxy) carbonyl)amino)-4-((3,5-bis(trifluoromethyl) benzyl) amino)-4-oxobutanoate (**3**): Fmoc-Asp-OtBu-OH (**1**) (90 mg, 0.22 mmol, 1.0 eq.), 3,5-bis(trifluoromethyl)benzylamine (**2**) (60 mg, 0.24 mmol, 1.0 eq.), EDC·HCl (40 mg, 0.25 mmol, 1.0 eq.) and HOBt (40 mg, 0.30 mmol, 1.2 eq.) were dissolved in anhydrous dimethylforamide (2 mL) and stirred at 0 °C for 2 hours under argon, and stirred for 1 hour at ambient temperature. The resulting mixture was then concentrated *in vacuo*, the crude product was dissolved in ethyl acetate (10 mL) before being successively extracted with sat. sodium carbonate (3 x 10 mL), aqueous citric acid (10% w/v solution, 3 x 10 mL), and brine (3 x 10 mL). The organic phase was dried with magnesium sulfate filtered and concentrated *in vacuo* to give the desired product as a white solid. Mp. 98-101 °C. IR V_{max} (Nujol)/cm^{-1} 3286 (N-H), 1726 (C=O), 1686 (C=O), 1659 (C=O). ^{1}H NMR (400 MHz, $CDCl_3$) δ 1.46 (s, 9H, -C(CH_3)$_3$), 2.6 (dd, J = 16.8, 6.4 Hz, 1H), 3.0 (dd, J = 17.2, 3.6 Hz, 1H), 4.23 (t, J = 7.2 Hz, 1H), 4.49-4.51 (m, 5H), 6.0 (bs, 1H), 6.7 (bs, 1H), 7.28 (m, 2H), 7.4-7.42 (m, 2H), 7.8 (d, J = 7.6 Hz, 2H), 7.76-7.81 (m, 5H). ^{13}C NMR (100 MHz, $(CD_3)_2SO$) δ 28.1 (-C(CH$_3$)$_3$), 37.7 (CH_2), 42.0 (CH_2), 47.1 (CH), 52.1 (CH), 66.2 (CH_2), 80.6 (-C(CH_3)$_3$), 95.7 (CF_3), 120.6 (Ar-CH), 120.9 (Ar-CH), 122.5 (Ar-Cq), 125.2 (Ar-Cq), 125.7 (Ar-CH), 127.5 (Ar-CH), 128.1 (Ar-CH), 128.4 (Ar-CH), 130.6 (q, J = 33 Hz, Ar-Cq), 156.3 (C=O), 169.8 (C=O), 171.4 (C=O). ^{19}F NMR (376 MHz, $CDCl_3$) δ -62.79 (s, 6F). HRMS (ESI) required for $C_{32}H_{30}F_6N_2O_5Na^+$ 659.1957 observed MNa^+ 659.1942. HRMS (ESI) required for $C_{32}H_{31}F_6N_2O_5^+$ 637.2092 observed MH^+ 637.2130.

(S)-3-((((9H-Fluoren-9-yl) methoxy) carbonyl) amino)-4-((3,5-bis (trifluoromethyl) benzyl)amino)-4-oxobutanoic acid (**4**): Ester (**14**) (100 mg, 0.16 mmol, 1.0 eq.) was dissolved in trifluoroacetic acid (2.0 mL, 26 mmol) and stirred for 2 hours at ambient temperature, before being concentrated in vacuo to yield the desired product as a white solid. Mp. 216-219

˚C. IR V_{max} (Nujol)/cm^{-1} 3266 (O-H), 1724 (C=O), 1655 (C=O), 1547 (N-H), 1376, 1293, 1175, 1126. ^{1}H NMR (400 MHz, MeOD) δ 2.74 (dd 1H, CH_2COOH J = 8.0 Hz), 2.87 (dd, 1H, CH_2COOH, J = 8.0 Hz), 4.22 (t, 1H, C*H*CH$_2$COOH, *J* = 8.0 Hz), 4.30-4.45 (m, 2H, CHCH_2 Fmoc), 4.46-4.62 (m, 3H, C*H*CH$_2$ Fmoc, CH_2 Bnz), 7.29 (t, 2H, *J* = 8.0 Hz, Fmoc 2 Ar-H), 7.37 (t, 2H, *J* = 8.0 Hz, Fmoc 2 Ar-H), 7.65 (d, 2H, Fmoc 2 Ar-H), 7.76-7.83 (m, 3H, Bnz Ar-*H*), 7.90 (d, 2H, Fmoc 2 Ar-H, *J* = 4.0 Hz). ^{13}C NMR (100 MHz, $(CD_3)_2SO$) δ 36.5 (CH_2), 42.0 (CH_2), 47.1 (CH), 52.05 (CH), 66.3 (CH_2), 95.7 (CF_3), 119.8 (Ar-Cq), 120.6 (Ar-CH), 120.8 (Ar-CH), 122.5 (Ar-Cq), 125.2 (Ar-Cq), 125.7 (Ar-CH), 127.5 (Ar-CH), 128.1 (Ar-CH), 128.2 (Ar-CH), 130.6 (q, *J* = 32 Hz, Ar-Cq), 156.4 (C=O), 171.8 (C=O), 172.2 (C=O). ^{19}F NMR (376 MHz, MeOD) δ -64.24 (s, 6F, 2 CF_3). HRMS (ESI) required for $C_{28}H_{23}F_6N_2O_5^+$ 581.1466 observed MH^+ 581.1506. HRMS (ESI) required for $C_{28}H_{22}F_6N_2O_5Na^+$ 603.4649 observed MNa^+ 603.1331. HRMS (ESI) required for $C_{28}H_{22}F_6N_2O_5^-$ 579.4677 observed MH^- 579.1335.

S3: General solid state peptide synthesis (SPPS) methodology

<u>General procedure 1 - Resin loading</u>

2-Chlorotrityl chloride resin was suspended in dichloromethane (2 mL) and very slowly stirred (to avoid resin grinding). Fmoc-AA-OH (1.2 eq.) was added followed by diisopropylethylamine (2.0 eq.); once no more HCl gas was observed the resin was stirred for 3 hours. Excess methanol (2 mL) was then added and the mixture stirred for a further 20 minutes. The resin was then filtered and washed with dimethylformamide (3 mL x 2), dichloromethane (5 mL x 2), hexane (5 mL x 2), dimethylformamide (3 mL x 2), dichloromethane (5 mL x 2) and finally hexane (5 mL x 2). The resin was then dried in vacuo and a sample removed for testing. Once tested, the resins were soaked overnight in dichloromethane / dimethylformamide (3 mL, 1:1).

General procedure 2 – Loading testing

Loaded resin (~10 mg) was stirred in piperidine/ dimethylformamide (3 mL, 2:8) for 2 hours, the absorbance at 290 nm was then measured indicating Fmoc presence, and therefore amino acid loading was estimated according to the Beer-Lambert equation. The remaining resin was transferred to a peptide synthesiser column [4].

General procedure 3 – Fmoc deprotection

The resin was washed by a steady flow of dimethylformamide, followed by a piperidine / dimethylformamide (2:8) mix until the absorbance at 290nm was reduced to starting point, and followed by an additional dimethylformamide wash.

General procedure 4 - Subsequent Fmoc-AA-OH

For all acid couplings, Fmoc-AA-OH (4.0 eq.), PyOxim (4.0 eq.), and diisopropylethylamine (4.5 eq.) in dimethylformamide (1.5 mL) were added to pre-treated resin in a peptide synthesiser column and stirred intermittently for 3 hours.

General procedure 5 – "Click" chemistry

The azide labelled substrate was slowly stirred in DMSO, water and acetonitrile (2:1:1, 1 mL). To this the Gd^{III}-DO3A alkyne was added (3 eq.) in DMSO (0.5 mL), followed by copper (II) sulfate hexahydrate (1 eq.) and sodium ascorbate (1 eq.). The suspension was slowly stirred for 20 hours.

General procedure 6 – "Click" washings

The resin bound product was successively washed with water, methanol, acetonitrile and dichloromethane.

General procedure 7 – Cleavage

The loaded resin was then washed with cleavage mixture (TFA: TIPS: H_2O, 9: 0.5: 0.5, 20 mL) and the solution slowly stirred for 6 hours before being concentrated in vacuo. The cleaved mixture was then washed with diethyl ether and filtered. The collected peptide was then dried in vacuo.

General procedure 8 – HPLC

Reversed-phase HPLC starting with eluent 100% A, increasing B to 90% to 25 minutes on a gradient, followed by 95% B at 27 minutes. The chromatogram was monitored at 215 nm.

Probe data

^{19}F NMR/MRI broad-range probe (substrate sequence- PLGLWAR); retention time 11.3 minutes, HRMS (ESI) observed MH^{2+} 921.8118 (40%), 922.3961 (74%), 922.9092 (84%), 923.4510 (100%), 923.9075 (69%), 924.4067 (49%).

^{19}F NMR/MRI MMP-2/-9 probe (substrate sequence- PLGMWSR); retention time 11.1 minutes, HRMS (ESI) observed MH^{2+} 938.6716 (34%), 939.1606 (71%), 939.6497 (83%), 940.1388 (100%), 940.6282 (61%), 941.1608 (45%).

S4: References

1. Knight CG, Willenbrock F, Murphy G. A Novel Coumarin-Labeled Peptide For Sensitive Continuous Assays Of The Matrix Metalloproteinases. FEBS J. 1992;296(3):263-6. doi: 10.1016/0014-5793(92)80300-6. PubMed PMID: WOS:A1992HC98000007.
2. Netzelarnett S, Mallya SK, Nagase H, Birkedalhansen H, Vanwart HE. Continuously Recording Fluorescent Assays Optimized For 5 Human Matrix Metalloproteinases. Anal Biochem. 1991;195(1):86-92. doi: 10.1016/0003-2697(91)90299-9. PubMed PMID: WOS:A1991FL67500014.
3. Gottlieb HE, Kotlyar V, Nudelman A. NMR chemical shifts of common laboratory solvents as trace impurities. J Org Chem. 1997;62(21). doi: 10.1021/jo971176v. PubMed PMID: WOS:A1997YC65700083.
4. White WCCaPD. Fmoc Solid Phase Peptide Synthesis: A Practical Approach. Hames BD, editor: Oxford University Press; 1999.
